# Neoadjuvant immunotherapy for early-stage hepatocellular carcinoma: the arts and science

**DOI:** 10.1016/j.esmogo.2023.08.001

**Published:** 2023-11-07

**Authors:** L.L. Chan, J.W.C. Kung, S.L. Chan

**Affiliations:** 1 State Key Laboratory of Translational Oncology, Department of Clinical Oncology, Sir YK Pao Centre for Cancer, Hong Kong, China; 2 Department of Surgery, Prince of Wales Hospital, The Chinese University of Hong Kong, Hong Kong, China; 3 Department of Clinical Oncology, Prince of Wales Hospital, The Chinese University of Hong Kong, Hong Kong, China

**Keywords:** liver neoplasm, ablation, surgery, systemic therapy, perioperative, hepatitis

## Abstract

Since the introduction of immune checkpoint inhibitors (ICI) a few years ago, we have witnessed unprecedented improvement in survival in hepatocellular carcinoma (HCC). Advanced stage HCC now has a median overall survival (OS) of >1.5 years compared to just a little more than 6 months a decade ago. In contrast, survival of early-stage HCC has made little progress due to the lack of effective adjuvant strategy, as recurrence after curative treatment can reach up to 70% at 5 years. Given the success of immunotherapy in advanced stage HCC, there is a growing interest in incorporating immunotherapy in the management of early-stage HCC. Recently, the IMBRAVE050 trial reported positive outcomes showing, for the first time, the use of adjuvant immunotherapy (e.g. atezolizumab), plus bevacizumab, is effective in prolonging recurrence-free survival in early-stage HCC following curative treatment. On the other end of the spectrum, there is an increasing momentum to explore neoadjuvant immunotherapy for early-stage HCC. Preclinical models have shown that neoadjuvant immunotherapy can effectively stimulate a broader range of T cells that can translate into a stronger anti-tumour immune response when the tumour is left *in situ*. Neoadjuvant immunotherapy has also been shown to effectively improve pathological complete response rates and prolong survival in other cancer types. Under this context, several small-scale, early phase trials have demonstrated promising results using neoadjuvant immunotherapy in early-stage HCC. In this mini review, we will discuss the rationale behind, currently available data, and considerations of study design on evaluating neoadjuvant immunotherapy in early-stage HCC.

## Highlights

• Early single-arm studies have shown promising results on neoadjuvant immunotherapy in early-stage HCC.
• Major pathological response post-neoadjuvant immunotherapy correlates with long-term survival in HCC.
• Randomized trials with well-defined eligibility criteria are needed to study the benefits of neoadjuvant immunotherapy.

## Introduction

For hepatocellular carcinoma (HCC), there is no doubt that the use of immune checkpoint inhibitors (ICIs) has revolutionized the treatment paradigm. Three ICI-based regimens have shown superior overall survival (OS) than sorafenib and become the standard first-line treatment for advanced HCC.^1^, ^2^, ^3^ Ongoing studies are also evaluating the combination of ICIs with locoregional therapy in intermediate-stage HCC. For early-stage HCC, surgery or ablation is conventionally recommended to achieve a cure of cancer and there is minimal role of systemic ‘palliative’ therapy. However, it is evident that recurrence is frequent even after treatment of curative intent, with 5-year recurrence rate following resection of up to 70%.^4^, ^5^, ^6^ Therefore there is a growing interest in incorporating immunotherapy in the management of early-stage HCC to improve outcomes of patients.

For resectable HCC, ICI-based treatment could be administered following primary curative treatment, with an aim to reduce or delay recurrences (i.e. adjuvant treatment). This approach is currently evaluated by a number of phase III clinical trials comparing ICI-based regimen with placebo/surveillance. Early findings of the IMBRAVE050 study recently reported an improvement of recurrence-free survival with the use of atezolizumab and bevacizumab.^7^ More mature results on both recurrence-free survival and OS from the IMBRAVE050 and other phase III clinical trials help clarify the benefits of adjuvant ICI. Neoadjuvant immunotherapy/ICI is another approach that is gaining interest. Compared with the adjuvant approach, neoadjuvant immunotherapy is less extensively studied in clinical trials but potentially more impactful in resectable HCC. In this review, we will discuss the rationale behind, currently available data, and consideration of study design on neoadjuvant treatment with immunotherapy in early-stage HCC.

## Rationale of neoadjuvant immunotherapy in cancer

The concept of neoadjuvant immunotherapy has already been confirmed to be clinically meaningful and feasible in other cancer types.^8^, ^9^, ^10^ Scientifically, preclinical studies have shown that the presence of intact tumour acts as a cancer vaccine *in situ*, which leads to the activation of a diverse repertoire of T cells when treated with ICI, resulting in a more robust antitumour response.^11^ In clinical trials, it has also been shown that neoadjuvant immunotherapy induces a broader T-cell expansion, higher rates of T-cell infiltrates into the tumour, and longer survival. For instance, in a phase II trial comparing the use of neoadjuvant–adjuvant nivolumab with nivolumab alone in advanced resectable melanoma, patients treated by the neoadjuvant approach had a significant improvement of event-free survival (EFS) at 2 years compared with the adjuvant arm (72% versus 49%).^12^ In the CHECKMATE-816 trial, which compared neoadjuvant nivolumab in combination with chemotherapy with neoadjuvant chemotherapy alone in resectable non-small-cell lung cancer, patients receiving neoadjuvant nivolumab–chemotherapy had a longer median EFS (31.6 versus 20.8 months; *P* = 0.005) and higher rates of pathological complete response (pCR; 24.0% versus 2.2%; *P* < 0.001).^13^ In stage II and III triple-negative breast cancer, the KEYNOTE-522 trial had again shown that the neoadjuvant immunotherapy–chemotherapy combination improved EFS (36 months: 84.5% versus 76.8%; *P* < 0.001) and pCR (64.8% versus 51.2%; *P* < 0.001).^14^^,^^15^

## Rationale of neoadjuvant immunotherapy in resectable HCC

**Figure 1:**
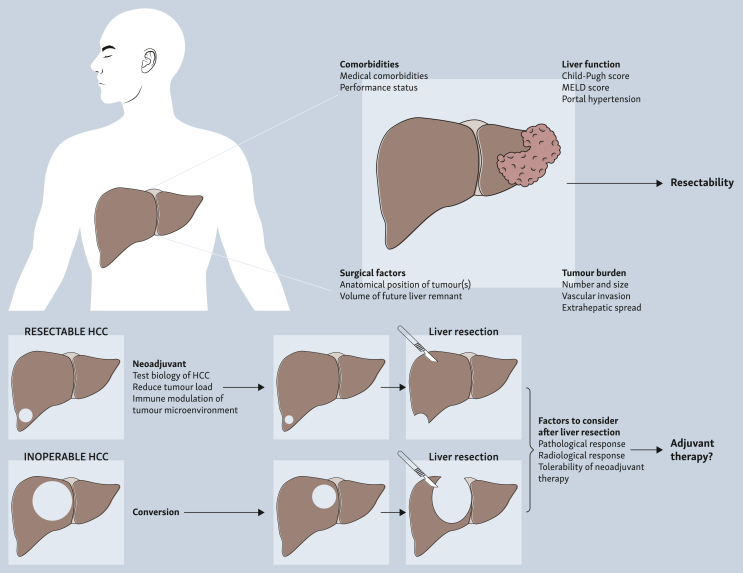
**Factors determining resectability of hepatocellular carcinoma (HCC) and biological rationale of neoadjuvant immunotherapy.** Patients’ factors such as medical comorbidities, liver function, surgical factors, and tumour burden are major determining factors of HCC resectability. Neoadjuvant immunotherapy can test the biology of HCC, reduce tumour load, modulate the tumour microenvironment, and may convert a previously inoperable HCC to an operable one. The response and tolerability to neoadjuvant immunotherapy may then guide the decision of whether further adjuvant therapy is needed. MELD, Model for End-stage Liver Disease.

The approach of neoadjuvant immunotherapy may have additional advantages in HCC. First, the decision on whether a tumour is resectable considers a combination of well-defined factors including tumour burden, liver function, volume of future liver remnant, portal hypertension, performance status, and comorbidities of the patient^16^^,^^17^ (Figure 1). However, the aggressive nature of disease and the high risk of recurrence in HCC still has posed challenges in selecting the suitable patients for curative surgery. The use of neoadjuvant immunotherapy could test the biology of the HCC prior to surgery or ablation. On the one hand, for patients whose disease progresses on neoadjuvant immunotherapy, liver resection might not provide the anticipated survival benefit and early recurrence can be expected after hepatectomy. On the other hand, with intratumour transcriptomic and immune heterogeneity,^18^^,^^19^ neoadjuvant immunotherapy could select out resistant tumours that do not achieve pathological response, and could inform a more effective and targeted adjuvant regime. Second, neoadjuvant immunotherapy could reduce tumour load and switch a borderline resectable tumour into a resectable one. The resultant liver resection could be less extensive, less technically challenging, and the overall postoperative risks could be reduced. The use of neoadjuvant treatment as conversion therapy to render an initially unresectable tumour to become amenable to surgery is however beyond the scope of this review. Third, the addition of locoregional therapy to neoadjuvant immunotherapy might have an immune-modulation effect on the tumour microenvironment, transforming the inherent immunosuppressive nature of the liver microenvironment into an immune-supportive niche, in which immunotherapy might be more effective^20^ (Figure 1).

## Existing data and ongoing studies on neoadjuvant treatment in HCC

Early-phase studies are now exploring the use of neoadjuvant (or perioperative) immunotherapy in upfront resectable or borderline resectable HCC (Table 1).^21^, ^22^, ^23^, ^24^, ^25^ The first study of neoadjuvant immunotherapy for HCC was a phase Ib trial that evaluated neoadjuvant cabozantinib and nivolumab in locally advanced disease where upfront resection was not recommended due to the presence of high-risk features such as multinodularity, portal vein invasion, or infiltrative disease.^21^ In this study of 15 patients, the neoadjuvant approach facilitated successful resections in 12 patients, including 5 patients who had major pathological response (>90% necrosis) to the neoadjuvant treatment. In another phase II study using neoadjuvant nivolumab or nivolumab–ipilimumab for 20 patients with upfront resectable disease, major pathological response (>70% necrosis) was seen in 30% (three in the nivolumab group and three in the nivolumab–ipilimumab) of patients.^22^ Interestingly, no radiological response was observed prior to surgery in patients who received neoadjuvant nivolumab–ipilimumab. However, one patient had complete pathological response despite tumour enlargement observed on preoperative magnetic resonance imaging, which was determined to be due to immune-cell infiltration.^22^ Furthermore, after a median follow-up of 26.8 months, none (0/6) of the patients who achieved major pathological response developed recurrence, while 7/14 patients who did not achieve major pathological response developed recurrence. In another phase II trial that evaluated neoadjuvant cemiplimab [anti-programmed cell death protein 1 (PD-1)] for 21 patients with upfront resectable HCC, 20% of the patients achieved significant tumour necrosis (>70% necrosis) and 15% of the patients had pCR.^23^ In all these trials testing neoadjuvant immunotherapy, grade 3 or above treatment-related adverse events (TRAEs) were in the range of 10%-30%, predominantly due to increased liver enzymes which are consistent with early trials testing immunotherapy in HCC.^2^^,^^26^ Given the promises that neoadjuvant immunotherapy holds for resectable and borderline resectable HCC, a number of early-phase trials are ongoing testing different neoadjuvant approaches, including the addition of other locoregional treatments such as external beam radiotherapy or internal radiation (Table 2).

**Table 1:** Current evidence on the role of neoadjuvant immunotherapy for resectable or borderline resectable hepatocellular carcinoma

Author (year)	Number recruited	Study phase	Disease burden	Neoadjuvant treatment	Neoadjuvant immunotherapy treatment schedule	Definition of pathological response	ORR (RECIST version 1.1)	Major pathological response	Pathological complete response	Grade 3 or above treatment-related adverse events
Ho et al. (2021)^21^	15	Ib	Borderline resectable	Cabozantinib + Nivo	240 mg Nivo Q2w for four cycles	>90% necrosis	7%	42%	8.3%	13.3%
Kaseb et al. (2022)^22^	27	II	Upfront resectable	Perioperative Nivo: 13 Nivo + ipi: 14	240 mg Nivo Q2w for three cycles; concurrent ipi 1 mg/kg for one dose with first dose of Nivo	>70% necrosis	Nivo: 23% Nivo + ipi: 0%	Nivo: 33% Nivo + ipi: 27%	25%	Nivo: 23% Nivo + ipi: 43%
Marron et al. (2022)^23^	21	II	Upfront resectable	Perioperative cemiplimab	350 mg cemiplimab Q3w for two cycles	>70% necrosis	15%	20%	15%	10%
Xia et al. (2022)^24^	18	II	Upfront resectable	Perioperative camrelizumab plus apatinib	200 mg camrelizumab Q2w for three cycles	>90% necrosis	16.7%	17.6%	5.9%	16.7%
Guo et al. (2023)^25^	61	II	Borderline resectable	Preoperative TACE plus sintilimab	200 mg sintilimab Q3w up to three cycles	>90% necrosis	—	49%	14%	28%

ipi, ipilimumab; Nivo, nivolumab; ORR, objective response rate; Q2w, every 2 weeks; Q3w, every 3 weeks; TACE, transarterial chemoembolization.

**Table 2:** Ongoing trials testing neoadjuvant immunotherapy for resectable or borderline resectable HCC

Study	Phase	Location	Neoadjuvant regime
PRIME-HCC [NCT03682276]	I/II	United Kingdom	Nivolumab 3 mg/kg Q3w for two cycles, plus ipilimumab 1 mg/kg Q6w for one cycle
NCT03510871	II	Taiwan	Nivolumab 3 mg/kg plus ipilimumab 1 mg/kg Q3w for four cycles
NCT05471674	II	Hong Kong	Nivolumab 3 mg/kg Q2w for three cycles
NEOTOMA [NCT05440864]	II	Canada	Tremelimumab 300 mg at cycle 1 plus durvalumab 1500 mg at cycle 1 and cycle 2 Q4w
NCT05185531	Ib	China	Tislelizumab Q3w for two cycles, plus SBRT 8Gy x 3 on day 1, 3 and 5
NCT05137899	II	Canada	Atezolizumab 1200 mg and bevacizumab 15 mg/kg Q3w for four cycles
NeoLeap-HCC [NCT05389527]	II	China	Pembrolizumab 200 mg Q3w for three cycles and lenvatinib 8 mg/12 mg for 9 weeks
NCT05701488	I	United States	Arm A: Durvalumab at cycles 1 and 2 Q4w, plus tremelimumab at cycle 1 Arm B: SIRT on day 1 followed by durvalumab at cycles 1 and 2 starting on day 3, plus tremelimumab at cycle 1 on day 3
NCT03867370	I/II	China	Arm A: Toripalimab 480 mg for one dose Arm B and C: Toripalimab 480 mg for one dose plus lenvatinib 8/12 mg
NCT04857684	I	United States	SBRT plus atezolizumab 1200 mg and bevacizumab 15 mg/kg Q3w for two cycles
NCT04123379	II	United States	Cohort C: nivolumab Q4w for two cycles Cohort D: nivolumab Q4w for two cycles plus CCR2/5 inhibitor (BMS-813160) 300 mg b.i.d for 28 days Cohort E: nivolumab Q4w for two cycles plus anti-IL-8 (BMS-986253) 2400 mg once

HCC, hepatocellular carcinoma; IL, interleukin; Q2w, every 2 weeks; Q3w, every 3 weeks; Q4w, every 4 weeks; SBRT, stereotactic body radiation therapy; SIRT, selective internal radiation therapy.

## Future directions on patient selection and study design on neoadjuvant therapy

Current studies investigating neoadjuvant therapy in resectable HCCs include a broad spectrum of patients that satisfy the indications for resection. However, within those indications exist a heterogeneous group of patients with diverse etiologies of liver disease, variable background of liver function, and tumours of different sizes and locations. The selection criteria for neoadjuvant therapy with resectable HCC need to be more specific and clearly defined.

Current indications of liver resection for HCC differ according to guidelines.^17^ The European Association for the Study of the Liver (EASL) guidelines recommend liver resection only in well compensated patients with a Model for End-stage Liver Disease (MELD) score <10 and solitary lesion without vascular invasion.^27^ The American Association for the Study of the Liver (AASLD) guidelines recommend liver resection for all potentially resectable solitary tumours ≤5 cm, with or without vascular invasion, and multifocal tumours none >5 cm, in the absence of impaired liver function.^28^ However, the Asian Pacific Association for the Study of the Liver (APASL) guidelines consider the selection criteria for liver resection set out by the EASL and the AASLD to be too strict and unsuitable for the Asia Pacific region.^29^ The APASL guidelines hence extend the indications for resection to those tumours with vascular invasion and in patients with decompensated liver disease (Child–Pugh B).^29^ For patients fulfilling the resection criteria according to the AALSD or EASL guidelines (e.g. solitary tumour <5 cm; absence of vascular invasion, and MELD score <10), the risk of recurrence is relatively low and neoadjuvant ICI may not be associated with sizeable absolute benefits. Neoadjuvant ICI is preferred among those patients who are considered resectable but with higher disease burden, namely tumour size >5 cm, multifocal HCC, and presence of vascular invasion. Within this population, the benefits of neoadjuvant therapy are expected to be more clinically meaningful and more readily demonstrated by clinical trials.

Other considerations may include the following: for patients with small subcapsular, peripherally located lesions without background liver disease, the role of neoadjuvant therapy might be equivocal. On the contrary, for patients with resectable disease within transplant criteria, unless a donor graft is not available, the overall and disease-free survival for patients undergoing neoadjuvant therapy followed by liver resection must be at least noninferior or comparable to that of liver transplantation before the former approach were to be considered standard.

Randomized study is required to confirm the efficacy of neoadjuvant immunotherapy reported by single-arm studies. In our opinion, the experimental arm should consist of a clearly defined and reasonable period of neoadjuvant immunotherapy, usually lasting for 2-3 months, followed by reassessment by imaging and multidisciplinary teams for surgery. If locoregional therapy such as transarterial therapy or radiation is used, the timing and details of these treatments should be defined as much as possible in the protocol. Patients in the control arm should receive surgery. The use of adjuvant or post-operative therapy is not yet standard at this juncture but protocol may allow the use of post-operative therapy in patients without achievement of major pathological responses in the experimental arm. Similar to neoadjuvant studies in other cancers, primary end points should consider event-free or disease-free survival and major/complete pathological response, preferably both evaluated by a blinded independent review. OS should also be a key secondary end point. TRAEs during the neoadjuvant therapy and perioperative period should be recorded with documentation of the causation. To document any layover toxicity of neoadjuvant therapy, patients should also be observed for at least 30 days post-operatively for any TRAEs.

## Conclusions

With the promise of immunotherapy in the context of advanced HCC, it is hopeful that neoadjuvant immunotherapy might add to the armamentarium for the management of resectable HCCs. The neoadjuvant regime, however, should be tailored to individual patients with varying degrees of background liver disease and tumour burden. To develop an effective and safe neoadjuvant therapy for HCC, a multidisciplinary approach is crucial for the selection of patients, administration of therapy, monitoring of toxicity, perioperative preparation, review of pathology samples, and decision on post-operative management.
